# Trypanosoma cruzi Ikiakarora (TcIII) Draft Genome Sequence

**DOI:** 10.1128/MRA.00453-20

**Published:** 2020-07-02

**Authors:** Inmaculada Gómez, Alberto Rastrojo, Fabián Lorenzo-Díaz, Francisco José Sánchez-Luque, Francisco Macías, Begoña Aguado, Basilio Valladares, José María Requena, Manuel Carlos López, M. Carmen Thomas

**Affiliations:** a Instituto de Parasitología y Biomedicina López-Neyra, Consejo Superior de Investigaciones Científicas, PTS-Granada, Granada, Spain; b Centro de Biología Molecular Severo-Ochoa (CSIC-UAM), Consejo Superior de Investigaciones Científicas, Madrid, Spain; c Instituto Universitario de Enfermedades Tropicales y Salud Pública de Canarias, Universidad de La Laguna, La Laguna, Spain; University of California, Riverside

## Abstract

Trypanosoma cruzi shows a genetic diversity that has been associated with the variability of clinical manifestations, geographical distribution, and preferential parasite-vector interactions. In an effort to better understand this genetic variability, here, the draft genome of T. cruzi strain Ikiakarora (discrete typing unit TcIII), which has been associated with the sylvatic cycle, is reported.

## ANNOUNCEMENT

Trypanosoma cruzi is a protozoan parasite that causes Chagas disease, one of the biggest public health problems in Latin America, with more than 10,000 deaths annually (1). Currently, the dynamics of migration of people from countries of endemicity has favored the spreading of Chagas disease to the United States, Canada, Europe, and the western Pacific. (2–4). This parasite presents a high level of genetic variability, being classified into six discrete typing units (DTUs), TcI to TcVI (5). TcIII is concentrated in South America and is associated mainly with the sylvatic cycle and the terrestrial niche (6). Here, the draft genome of T. cruzi strain Ikiakarora (IRHO/CO/95), which belongs to DTU III as characterized by 24Sα rRNA, miniexon, and 18S rRNA markers, is reported (7). This strain was isolated in Catatumbo (North Santander, Colombia) from the sylvatic vector Rhodnius prolixus, the second most frequent transmitting vector of Chagas disease (8). Analyses of the H2A gene units of different strains isolated in Colombia showed that the genome of strain Ikiakarora has a high degree of plasticity (9).

Epimastigote forms were cultivated at 28°C in liver infusion tryptose (LIT) medium supplemented with 10% fetal bovine serum. When the parasites reached log phase (10 × 10^6^ to 20 × 10^6^ parasites/ml), they were collected and lysed in 1% NP-40. Nuclei were lysed by addition of 1% SDS, and genomic DNA was purified by phenol-chloroform extraction and ethanol precipitation. Sequencing was carried out using Ion Torrent technology (Thermo Fischer Scientific, Inc.). Library construction, size selection, quality filtering, DNA concentration, and processing were performed as previously described (10), obtaining 3,928,712 raw reads (average read length, 254 bp).

Sequence reads were analyzed using FastQC v0.10.1 (default settings) (http://www.bioinformatics.babraham.ac.uk/projects/fastqc), and Prinseq v0.20.4 (11) was used iteratively for quality filtering using the following parameters: -derep 14, -ns_max_p 1 -ns_max_n 3 -trim_ns_left 1 -trim_ns_right 1, -trim_qual_right 20 -trim_qual_type mean -trim_qual_window 5 -trim_qual_step 1, -trim_qual_right 20 -trim_qual_type mean -trim_qual_window 1 -trim_qual_step 1, -trim_qual_left 20 -trim_qual_type mean -trim_qual_window 5 -trim_qual_step 1, -trim_qual_left 20 -trim_qual_type mean -trim_qual_window 1 -trim_qual_step 1, -lc_method entropy -lc_threshold 50, -min_qual_mean 25, and -min_len 50. The obtained 3,338,764 quality-filtered reads (average read length, 266 bp) were assembled into 11,096 contigs totaling 18,492,845 bp, with an *N*_50_ value of 2,193 bp and an average contig size of 1,666 bp using CLC Genomics Workbench software v8.0 (Qiagen) (length fraction, 0.90; similarity fraction, 0.97; minimum contig length, 500 bp). The longest contig was 33,607 bp, and the G+C content was 48.72%. To assess genome assembly completeness, BUSCO (Benchmarking Universal Single-Copy Orthologs) v4.0.5 analysis (12) (parameters: -m genome, -l euglenozoa_odb10) was performed on the assembled genome using the Euglenozoa odb10 orthologue set (*n* = 130). A total of 106 complete BUSCOs (81.6%) and 102 single-copy BUSCOs (78.5%) were identified from 130 BUSCO-searched groups.

### Data availability.

The T. cruzi Ikiakarora assembled genome has been deposited in GenBank (accession number WWPZ00000000), and the raw reads have been deposited in the SRA (accession numbers SRR11235102, SRR11235103, and SRR11235104 and BioProject accession number PRJNA595095).
